# Population-pharmacokinetic/pharmacodynamic model of *atractylodes lancea* (Thunb.) DC. administration in patients with advanced-stage intrahepatic cholangiocarcinoma: a dosage prediction

**DOI:** 10.1186/s12906-024-04618-8

**Published:** 2024-11-06

**Authors:** Teerachat Saeheng, Juntra Karbwang, Kesara Na-Bangchang

**Affiliations:** 1 Centre of Excellence in Pharmacology and Molecular Biology of Malaria and Cholangiocarcinoma, Chulabhorn International College of Medicine, Thammasat University (Rangsit Campus), 99, moo 18, Phaholyothin Road, Klongneung sub-district, Klongluang district, Pathumthani, 12121 Thailand; 2 Graduate Program in Bioclinical Sciences, Chulabhorn International College of Medicine, Thammasat University, Klongluang, Pathumthani 12120 Thailand; 3 Drug Discovery and Development Centre, Office of Advanced Science and Technology, Thammasat University (Rangsit Campus), Klongluang, Pathumthani Thailand

**Keywords:** Population-pharmacokinetic model, *Atractylodes Lancea*, Intrahepatic cholangiocarcinoma, Biliary tract cancer, Dose prediction, Clinical study

## Abstract

**Background:**

A recent phase 2A clinical study of *Atractylodes lancea* (Thunb.) DC. (AL) in patients with advanced-stage intrahepatic cholangiocarcinoma (iCCA) demonstrated significant reduction of the risk of tumor progression and mortality with a dose ranging from 1,000 to 2,000 mg. The present study aimed to determine the potential dosage regimen of AL for further phase 2B clinical study.

**Methods:**

Plasma-concentration time profiles of total AL bioactivity and clinical efficacy in patients with advanced-stage iCCA were obtained from Phase 2 A study. The population pharmacokinetic (pop-PK) model was developed. The pop-PK model and Monte-Carlo (MC) simulation, in conjunction with maximum concentration of AL (C_max_) as a cut-off criterion, was performed and validated with clinical data. The optimal model was used to simulate further dosage regimens and clinical efficacy of AL.

**Results:**

The pop-PK properties of total AL bioactivity were best described by a compartmental model with zero-order absorption (without delay) and linear clearance. None of the investigated covariates improved model accuracy.The developed pop-PK with MC simulations following once-daily dosing of 1,000 mg and 2,000 mg adequately predicted the clinical efficacy (tumor progression and mortality). The once-daily dose of 2,500 mg is recommended for further phase 2B clinical study due to its relatively high efficacy on tumor progression inhibition (73%) and mortality rate reduction (71%) without excessive number of the administered capsules (23 capsules) and low risk of toxicities (<5%).

**Conclusions:**

The applied pop-PK model with MC simulation, along with the appropriate cut-off pharmacokinetic parameters, can be used as a potential tool for supporting dosage prediction and selection for clinical studies, and thus reducing the rate of drug development failures.

**Trial registration:**

www.thaiclinicaltrials.org, WHO ICTRP search, TCTR20210129007, Registed 29 January 2021.

**Supplementary Information:**

The online version contains supplementary material available at 10.1186/s12906-024-04618-8.

## Background

Reducing the attrition of new molecular entities (NMEs) is crucial for clinical drug development. This process generally takes 10–15 years with an average cost of $1–2 billion *per* NME for clinical approval [1]. A recent study on clinical drug development failures during 2015–2017 revealed that the attrition rate of clinical failures was increased to 90%, 40–50% from the lack of clinical efficacy, 30% from unmanageable toxicities, 10–15% from poor drug-like properties, and 10% from others [2]. Most of the failures during phase II and III clinical trials were due to the lacks of efficacy (52%) and safety (24%) [2]. Of these, the highest percentages of clinical failures were in the oncology area (32%) [2]. Based on subgroup analysis, insufficient efficacy of candidate anticancer drugs remains a main reason for clinical failure in Phase II (48%) and Phase III (55%) [2]. High failure rates in clinical development due to insufficient efficacy suggest an inappropriate conventional approach for dose selection. Population pharmacokinetic/pharmacodynamic (pop-PK/PD) modelling mixed-effects with Monte Carlo (MC) simulation is one of the model-informed drug development (MIDD) approaches that is used as a tool for optimal dosage prediction for a clinical study [3, 4]. It has been recognized as an effective tool for supporting oncology drug development and regulatory review [4, 5]. The pop-PK/PD in conjunction with MCMC could be a valuable tool in guiding the oncology drug development in low-and-middle Income countries by reducing the time and cost of drug development.

Cholangiocarcinoma (CCA) is a burden disease with a poor prognosis and unsatisfactory therapy [6, 7]. The world highest incidence of CCA is reported from Northeastern Thailand [6]. The development of new therapeutic options is necessary to tackle this fatal disease. A recent phase 2 A clinical study of *Atractylodes lancea* (Thunb.) DC. (AL) in patients with advanced-stage intrahepatic cholangiocarcinoma (iCCA) revealed that AL significantly increased overall survival rate (OSR), disease control rate (DCR), and prolonged progression-free survival (PFS) compared with standard supportive care alone [8, 9]. The objective of this study was to determine the potential dosage regimen for a phase 2B clinical study.

## Materials and methods

### Data source and study population

The study was a retrospective clinical study, which was conducted at the Sakhon Na-Kon Hospital in the Sakhon Na-Kon, Thailand (*n* = 47) as part of a single-center, open-label, randomized, controlled phase 2 A trial (registration number TCTR20210129007, 04/01/2021, www.thaiclinicaltrials.org, WHO ICTRP search) [8]. The study protocol was approved by the Sakhon Na-Kon Hospital Ethics Committee (No. 049/2564). The study was conducted in compliance with guidelines for Good Clinical Practice (GCP) and the Helsinki Declaration. The inclusion and exclusion criteria had described in previous study [8]. The inform consent form have been received prior to participate in the study.

### Treatment

Patients who fulfilled the inclusion and had none of the exclusion criteria were randomized to receive treatment as follows:

- Group 1: once-daily dose of 1,000 mg of the capsule formulation of standardized AL extract (CMC-AL, 9 capsules each) for 90 days, in conjunction with standard supportive care (*n*=15).
- Group 2: once-daily dose of 1,000 mg CMC-AL (9 capsules each) for 14 days, followed by 1,500 mg (14 capsules each) for 14 days, and 2,000 mg (18 capsules each) for 62 days, in conjunction with standard supportive care (*n*=16).
- Group 3: standard supportive care alone (*n*=16) (control group).

A block randomization (three block size), allocation concealment, and blinding (participants and care providers) were applied. The number of patients in each arm are adequate for a phase-II A clinical trial as the number of participants in each group was at least 12 participants (minimal requirement).

### Pharmacokinetic study

The pharmacokinetic study was conducted in 32 patients. Blood samples were collected at 0, 0.25, 0.5, 1, 1.5, 2, 2.5, 3, 4, 5, 6, 8 h (5 ml each) from each patient on day 1 for group 1, and day 14 and day 28 for group 2.

### Population-pharmacokinetic (pop-PK) modelling

The pop-PK model was developed using nonlinear mixed-effects modelling (MonolixSuite software, version 2023R1, Antony, France; Lixoft SAS, 2023). The built-in stochastic approximation expectation-maximization algorithm was used to estimate pharmacokinetic parameters. Different compartment models with kinetic orders of the absorption and elimination processes were used to fit drug concentration-time data. The distribution of pharmacokinetic parameters was normal in a logarithmic scale.

### Monte-Carlo simulations

The final pop-PK (1000 virtual patients) model was used to simulate optimal dose regimens of CMC-AL that provided highest clinical efficacy (prevention of progressive disease and reduction of mortality) using Monte-Carlo (MC) simulations (Simulix version 2023R1, Antony, France; Lixoft SAS, 2023). The simulated dose regimens included once-daily dose regimens of 1,000, 1,500, 2,000, 2,500 and 3,000 mg. The simulated regimens are based on trials and errors until it provides the curative rate close to 100%.The period of the simulation was 3 months and 1 year.

### Effect of patients’ adherence

Due to the long course of treatment of CMC-AL in iCCA therapy (> 3 months), the effects of patients’ adherence to medication (i.e., 100%, 80%, 50%, and 20% adherence) on treatment efficacy were evaluated for the once-daily dose of 1,500, 2,000, 2,500, and 3,000 mg. The adherence to therapy with oral oncology drugs was reported to be ranged from 20 to 100% [10].

### Clinical efficacy

Inhibitory effects of CMC-AL on tumor progression and prevention of death were used as efficacy criteria.

### Criteria for optimal dosage regimens

The cut-off values for total AL bioactivity for inhibitory effects on tumor progression and death prevention were maximum concentration (C_max_) ≥ 32.39 and ≥ 21.42 mg/L, respectively [9].

### Validation of pop-PK model with Monte-Carlo simulations

The inhibitory effects on tumor progression and death prevention were used as clinical efficacy criteria for model validation. Progressive disease in groups 1, 2, and 3 were found in 11 (84.61%), 7 (47%) and 11 (84.61%) cases, respectively. The mortality rates in groups 1, 2, and 3 were found in 3 (71%), 3 (30%) and 12 (92%) cases, respectively. Predefined criteria for a model selection were: (i) objective function value (OFV), Akaike Information Criteria (AIC), Bayesian Information Criteria (BIC), and Corrected Bayesian Information Criteria (BICc), and (ii) percentage of root mean square errors (RSE%), graphical goodness of fit (GOF), i.e., observed *versus* predicted concentrations, scatter plot of residual, and virtual predictive check (VPC).

### Statistical analysis

Comparisons of clinical efficacy between once-daily dose of 1,000 mg and other regimens, and the effects of patietnts’ adherence to medication were performed using Chi-square test (two quantitative variables). Statistical significance level (odd ratios (OR) and Chi-square test) was set at α = 0.05.

## Results

**Fig. 1:**
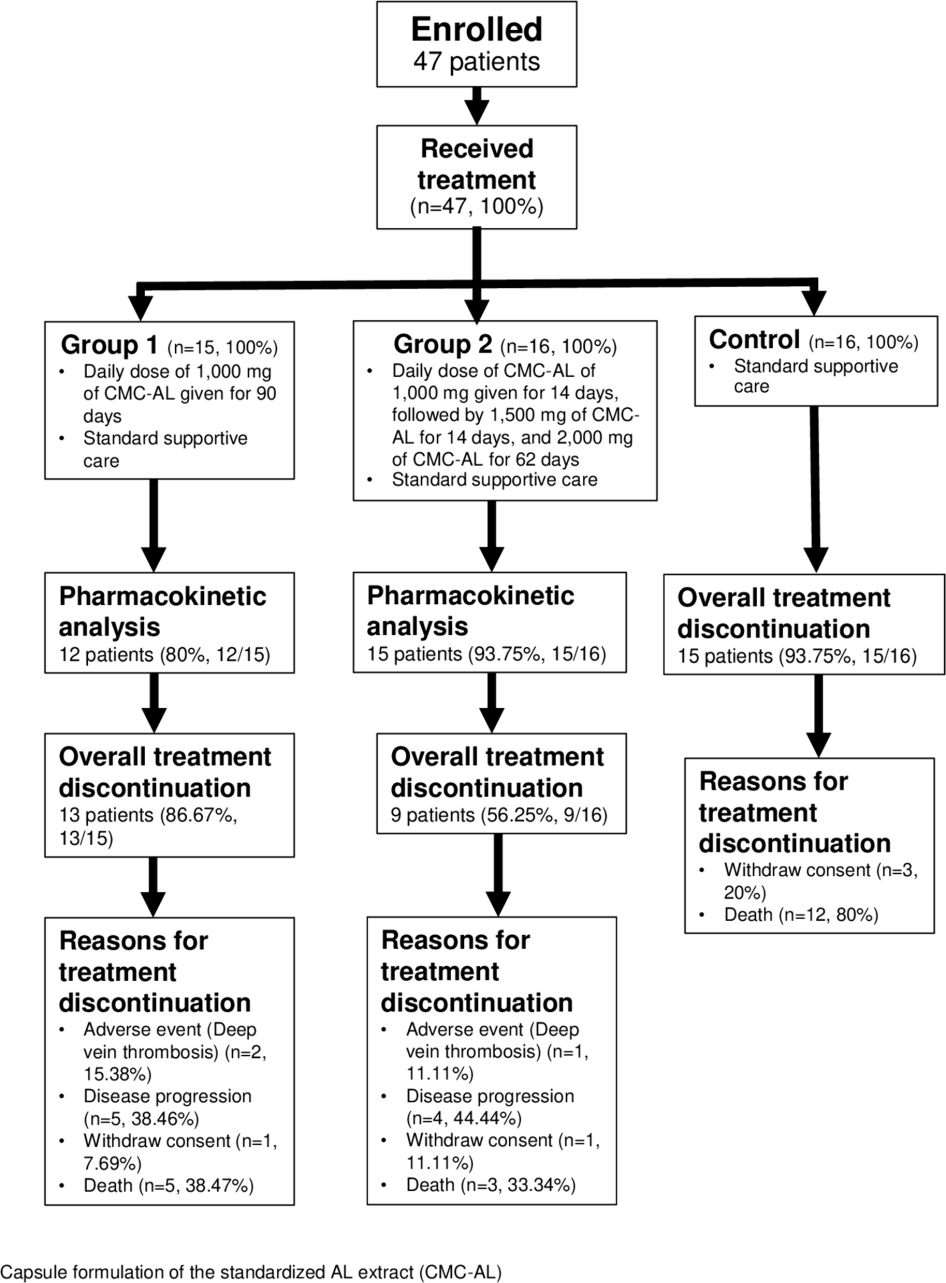
A flow chart of patients in the study

**Fig. 2:**
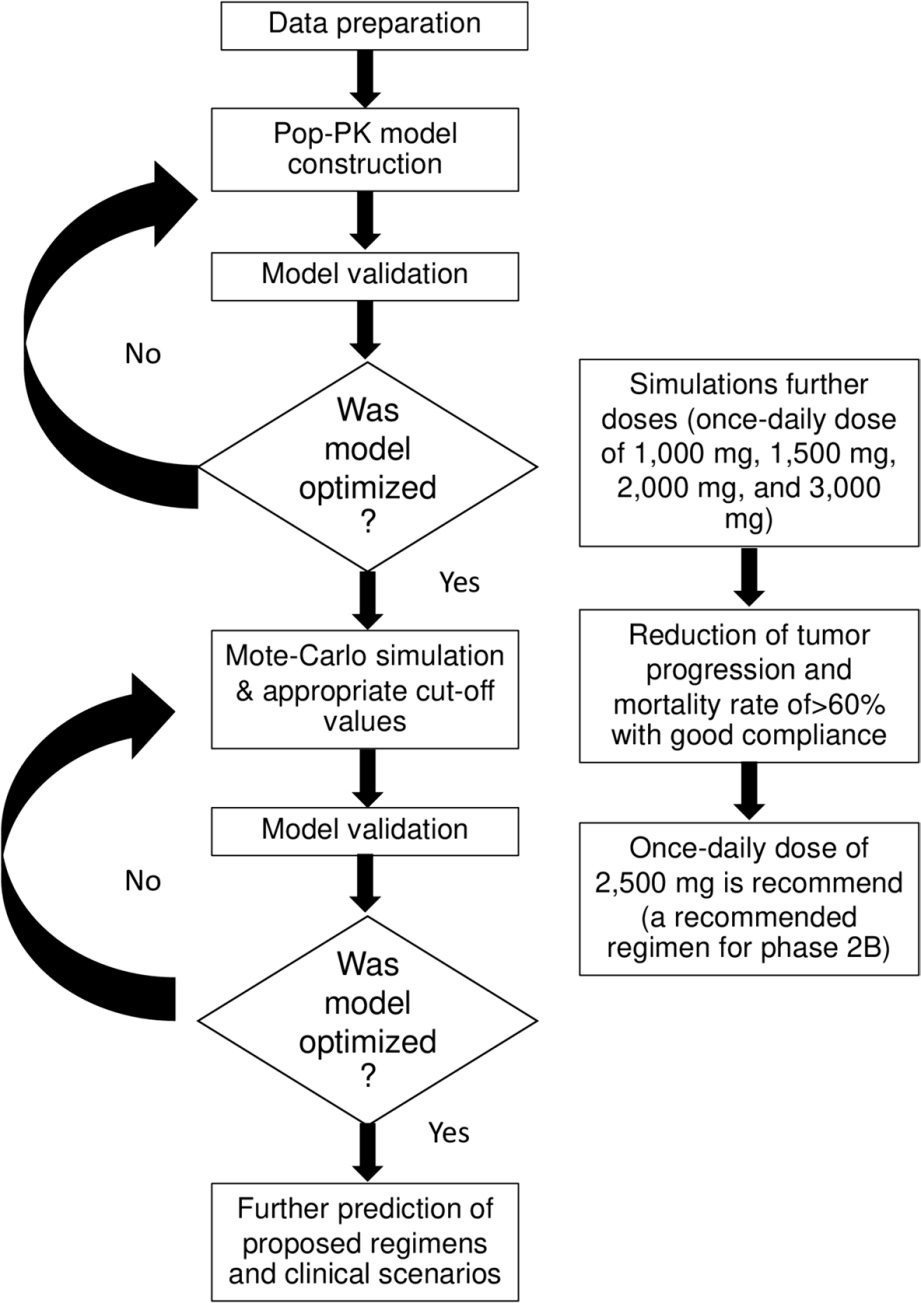
A schematic workflow and dose selection criteria

Fifteen, sixteen, and sixteen participants were assigned randomly for group 1, 2, and 3 respectively. One compartmental model with zero-order absorption (without delay) and linear clearance best characterized the pop-PK properties of total bioactivity of AL for group 1 patients on day 1 and group 2 patients on days 14 and 28. None of the investigated covariates (sex, age, weight, and height) improved the model. Final pop-PK parameters for group 1 (day 1), and group 2 day 14 annnd day 28 patients are shown in Tables 1 and 2, and 3, respectively. The variation of all parameters used were low (%RSE and SE, Tables 1, 2 and 3). The OFV, AIC, BIC, and BICc are shown in Table S1. The GOF Figs are illustrated in Fig S1-12. The study flow-chart and schematic workflow for dose selection are shown in Figs. 1 and 2, respectively. The Pop-PK model for atractylodin, the active constituent of AL, was developed; however, no significant association of the pharmacokinetic parameters of atractylodin and clinical efficacy was observed. Therefore, pop-PK model of atractylodin was not used for further optimal dose prediction.

**Table 1:** Pharmacokinetic parameters of total AL bioactivity in Group 1 (day 1)

Fixed effects				
	Value	%CV	S.E.	%RSE
**Tk_pop**	0.85	NA	0.15	17.3
**V_pop**	42.82	NA	4.86	11.3
**Cl_pop**	20.9	NA	3.36	16.1
**Standard deviation of Random effects**				
**Omega_Tk0**	0.51	54.88	0.14	27.2
**Omega_V**	13.2	30.82	4.25	32.2
**Omega_Cl**	0.54	57.91	0.12	21.8
**Error model parameters**				
**a**	0.36	NA	0.1	28.4
**b**	0.32	NA	0.038	11.8

**Table 2:** Pharmacokinetic parameters of total AL bioactivity in Group 2 (day 14)

Day 14				
Fixed effects				
	Value	%CV	S.E.	%RSE
**Tk_pop**	1.11	NA	0.12	11.2
**V_pop**	32.57	NA	2.9	8.89
**Cl_pop**	17.2	NA	2.03	11.8
**Standard deviation of Random effects**				
**Omega_Tk0**	0.35	36.57	0.093	26.3
**Omega_V**	8.89	27.3	2.44	27.4
**Omega_Cl**	0.44	46.6	0.087	19.5
**Error model parameters**				
**a**	1.39	NA	0.33	24.0
**b**	0.17	NA	0.087	19.3
**Day 28**				

**Table 3:** Pharmacokinetic parameters of total AL bioactivity in Group 2 (Day 28)

Fixed effects				
	Value	%CV	S.E.	%RSE
**Tk_pop**	0.95	NA	0.086	9.10
**V_pop**	32	NA	2.51	7.85
**Cl_pop**	16.13	NA	1.72	10.7
**Standard deviation of Random effects**				
**Omega_Tk0**	0.28	28.3	0.081	29.3
**Omega_V**	0.25	25.03	0.06	24.5
**Omega_Cl**	0.38	38.92	0.077	20.4
**Error model parameters**				
**a**	2.21	NA	0.43	19.2
**b**	0.084	NA	0.025	29.6

### Model validation

The developed pop-PK model in conjunction with MC simulation adequately predicted tumor progression and mortality in patients with advanced-stage iCCA in groups 1 and 2. The predicted tumor progression vs. observed clinical data in groups 1 and 2 patients were 96% vs. 85% and 55% vs. 47%, respectively. The predicted death rates vs. observed clinical data in groups 1 and 2 were 66% vs. 71%, and 29% vs. 30%, respectively.

### Tumor progression

In the absence of patients’ adherence effect (100% compliance) following a 3-month course, the proportions of patients with tumor progression following the once-daily dose regimens of 1,000, 1,500, 2,000, 2,500 and 3,000 mg were 96%, 83%, 55%, 27%, and 12%, respectively. The 1,000 and 1,500 mg daily dose regimens were suboptimal as the proportions of patients with tumor progression were high. Therefore, no further simulations of the effects of patients’ adherence were performed for both regimens. When the patient’s adherence was decreased to 80%, the proportions of patients with tumor progression following the once-daily dose regimens of 2,000, 2,500 and 3,000 mg were 63%, 43% and 30%, respectively. The corresponding values for the 50% adherence were 78%, 62% and 59%, respectively. With 20% adherence, the proportions of patients with tumor progression following the once-daily regimens of 2,000, 2,500 and 3,000 mg were 91%, 86% and 84%, respectively. When the administration period of CMC-AL was prolonged to 1 year, the proportions of patients with tumor progression with 100% adherence for all regimens remained unchange. However, the proportions of patients with tumor progression following the once-daily dose regimens of 2,000, 2,500 and 3,000 with 80% adherence were slightly increased to 65%, 44%, and 32%, respectively. The corresponding values for the 50% adherence were 82%, 61% and 53%, respectively, and those for the 20% adherence were 92%, 86% and 80%, respectively. Prolonged drug administration to 1 year did not affect tumor progression. In comparison with standard supportive care, the once-daily dose regimen of 2,000 mg [OR: 0.197 (0.04–0.91), *p* = 0.03, X^2^(4.961, 1)], 2,500 mg [OR: 0.082 (0.018–0.39), *p* < 0.001, X^2^ (14.61, 1)] and 3,000 mg [OR: 0.035 (0.007–0.39), *p* < 0.001, X^2^ (29.71, 1)] were associated with a lower risk of tumor progression. In contrast, there were no significant differences in tumor progression following the once-daily dose regimens of 1,000 mg [OR: 4.364 (0.7469–20.55), *p* = 0.09, X^2^ (2.966,1)] and 1,500 mg [OR: 1.12 (0.23–4.93), *p* = 0.89, X^2^ (0.018, 1)].

### Deaths

The proportions of death cases (mortality rate) with 100% drug adherence following the once-daily dose regimens of 1,000 mg, 1,500 mg, 2,000 mg, 2,500 mg and 3,000 mg were 66%, 50%, 29%, 12% and 3%, respectively. The once-daily dose regimen of 1,000 was suboptimal since the mortality rate exceeded 50%. Therefore, no additional simulations of the effect of this regimen on patients’ adherence was done. The mortality rates following the 1,500, 2,000, 2,500 and 3,000 mg with 80% adherence were 56%, 44%, 33% and 24%, respectively. The corresponding values for 50% adherence were 73%, 61%, 53% and 52%, respectively, while those for the 20% adherence were 89%, 88%, 82% and 80%, respectively. Similarly to tumor progression, the mortality rates in these regimens remained unchanged when the duration of administration was prolonged to 1 year. The proportions of death cases with 80% adherence following a 1-year once-daily regimens of 1,500 and 2,000 mg were increased to 60% and 49%, respectively. The mortality rates were slightly decreased to 32% and 20% following a 1-year once-daily regimens of 1,500 and 2,000 mg, respectively. With 50% adherence, the mortality rates were 75%, 62%, 53% and 52%, respectively. With 20% adherence, the proportions were 90%, 87%, 80% and 79%, respectively. In comparison with a standard supportive care, no significant difference was found between the once-daily regimen of 1,000 mg and standard supportive care [OR: 0.21 (0.013–2.289), *p* = 0.21, X^2^(1.56, 1)]. Significant differences were however, found with the once-daily regimens of 1,500 mg [OR: 0.083 (0.007-0.5), *p* = 0.004, X^2^(8.32, 1), 2,000 mg [OR: 0.03 (0.003–0.21), *p* < 0.001, X^2^(19.94, 1)], 2,500 mg [OR: 0.01 (0.001–0.08), *p* < 0.001, X^2^(44.35, 1)] and 3,000 mg [OR: 0.002 (0.0002–0.023), *p* < 0.001, X^2^(79.7,1)].

## Discussion

With %RSE of < 30%, the developed pop-PK model accurately described the pharmacokinetic characteristics of the total AL bioactivity. In addition, the developed pop-PK with MC simulations following the once-daily dose regimens of 1,000 mg and 2,000 mg adequately predicted the clinical efficacy (tumor progression and mortality), indicating the reliability of the developed model for the prediction of clinical efficacy for alternative dose regimens and scenarios. Furthermore, the extended period of drug administration had no effect on clinical efficacy.

### Tumor progression

**Fig. 3:**
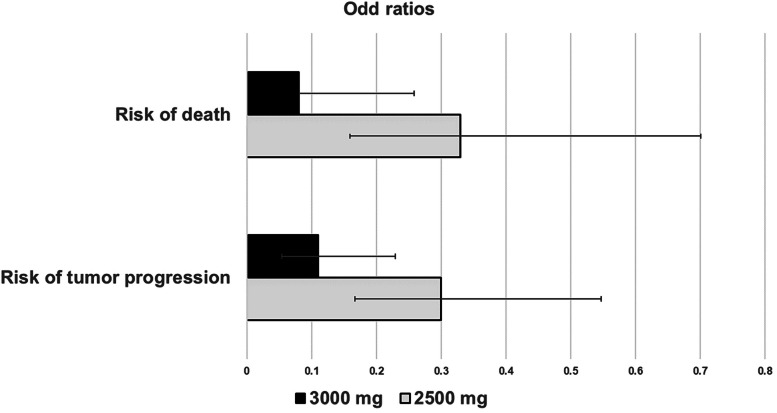
Comparisons of treatment’s efficacy (risk of tumor progression and death) between once-daily doses of 2,000 mg and other proposed regimens [Data are presented mean (± 95% confidence interval)]

In general, the once-daily dose regimens of 1,000 and 1,500 mg were inappropriate for the advanced -stage iCCA treatment since there were no significant differences between those regimens and standard supportive care. Therefore, the once-daily dose regimens of 2,000, 2,500, and 3,000 mg were suggested. Nevertheless, in comparison with 2,000 mg, the once-daily regimen of 3,000 mg [OR: 0.112 (0.054–0.229) X^2^(41.499,1), *p* < 0.001] showed the most potent inhibitory effect on tumor progression (tumor progression = 12%) (Fig. 3). This regimen is a potential dosage regimen for a phase 2B study. However, the total number of tablets is rather excessive (28 capsules total), which may influence the patient’s adherence. It is evident that the percentage of patients with tumor progression following the once-daily regimen of 3,000 mg with 20% adherence was increased to 80%. The once-daily regimen of 2,500 mg is, therefore, recommended (23 capsules) [OR: 0.33 (0.159–0.701), X^2^ (16.205, 1), *p* < 0.001]. This regimen with 100% of adherence showed the inhibitory effect of tumor progression over 70%. Notably, the proportions of patients with tumor progression for all regimens were higher than 80% when the patient’s adherence was decreased to 20%. Therefore, the patient’s adherence is a crucial factor for the success of treatment. Hirao and colleagues reported that the number of oral chemotherapy medications given daily (*p* = 0.04) and the frequency of drug administration (*p* = 0.01) influences the treatment course and clincal efficacy due to non-adherence of the patients to medication [11]. Therefore, the once-daily dose of 2,500 mg with 23 capsules would reduce the risk of non-adherence compared to 3,000 mg. This daily-dose regimen is approximately the maximum recommended starting dose (MRSD) of 2,400 mg [12] and the risk of bleeding as previously reported in an in vitro experiment is unlikely [13]. The lower once-daily dose of 2,000 mg did not produce toxicity [8]. In addition, the predicted proportion of hematological toxicities based on the modelling approach following the once-daily dose of 2,500 mg was less than 5% [14], suggesting a low risk of hematological toxicity. Nevertheless, monitoring of blood coagulation profiles in patients receiving AL are advised in further clinical studies.

Gemcitabine plus cisplatin (first-line therapy) for advanced-stage biliary tract cancer has been reported to reduce the risk of tumor progression compared to gemcitabine alone (HR: 0.63 (0.51–0.77), *p* < 0.001) [15]. Regorafenib (a targeted therapy) for advanced-stage biliary tract cancer inhibited tumor progression compared with placebo (HR: 0.49 (0.29–0.81), *p* = 0.004) [16]. A recent study of ivosidenib for refractory CCA therapy revealed that ivosidenib also prevented tumor progression compared with placebo (HR: 0.37 (0.25–0.54), *p* < 0.0001) [17]. Besides targeted therapy, a recent immunotherapy study revealed that patients treated with durvalumab in combination with gemcitabine/cisplatin in patients with advanced-stage CCA had a significant reduction in the risk of disease progression (HR: 0.75 (0.63–0.89), *p* = 0.001) compared with gemcitabine/cisplatin alone [18]. In comparison with standard supportive care, CMC-AL at the once-daily dose regimens of 2,000 mg, 2,500 mg, and 3,000 mg showed superior inhibitory effects on tumor progression compared with regorafenib [0.2 (NNT = 3.06) vs. 0.08 (NNT = 1.865) vs. 0.035 (NNT = 1.45) vs. 0.49, respectively]. With those regimens, the maximal number-need to be treated was only 3 persons. This suggests that progression-free disease is expected to be observed in every three patients who received treatment with those regimens. Association between the risk of severe adverse effects and toxicities following conventional and targeted chemotherapies and compliance to medications have been well demonstrated [19], which could result in ineffective treatment or treatment failure. In the absence of significant adverse effects, AL would be a preferable alternative to the currently available medications.

### Deaths

The once-daily regimen of 1,000 mg was ineffective for advanced-stage iCCA in reducing the mortality rate compared with standard supportive care. The remaining dose regimens were effective. However, the proportion of deaths following once-daily regimen of 1,500 mg was high (50%). This regimen was inappropriate. The once-daily dose regimens of 2,000, 2,500 and 3,000 mg were recommended. In comparison with 2,000 mg, the once-daily dose regimen of 3,000 mg [OR: 0.076 (0.022–0.258), X^2^(25.149, 1), *p* < 0.001] provided the lowest mortality rate among the remaining regimens, and is therefore recommended for further phase 2B clinical study (Fig. 3). Nevertheless, the highest number of capsules administration (28 capsules) may influence patients’ adherence and compromise treatment efficacy. The once-daily regimen of 2,000 mg (18 capsules) or 2,500 mg (23 capsules total) may be more practical. It was noted that the proportions of death cases following both regimens were lower than 30% (29% and 12%, respectively). The once-daily dose of 2,500 mg reduced the risk of death by 3-fold compared with the once-daily dose of 2,000 mg (Fig. 3). The proportion of death cases with 50% adherence to the once-daily regimen of 2,000 mg was close to 50%. The once-daily regimen of 2,500 mg is therefore a better option as the mortality rate was relatively lower (32%). Similarly to the effective dose for inhibition of tumor progression, the mortality rates for all regimens exceeded 80% when the patient’s adherence dropped below 20%. In case of poor tolerance to medication, the once-daily dose regimen of 2,000 mg is an alternative regimen.

It has been demonstrated that gemcitabine/cisplatin combination therapy reduces mortality risk (HR: 0.64; 95%CI: 0.52 to 0.80; *p* < 0.001) compared with gemcitabine alone [15]. In contrast, capecitabine did not reduce mortality risk in patients with advanced-stage CCA (HR: 0.81, 95%CI: 0.63–1.04, *p* > 0.05) [20]. FOLFOX regimen containing folinic acid, fluorouracil and oxaliplatin was reported to reduce the risk of death (HR: 0.69 (0.5–0.97), *p* = 0.031) compared with control [21]. In addition, combination of durvalumab and gemcitabine/cisplatin was reported to reduce the mortality risk (HR: 0.80 (0.66–0.97), *p* = 0.021) compared with gemcitabine/cisplatin [18]. The once-daily regimens of 2,000 and 2,500 mg are superior to the FOLFOX regimen with regard to the reducing effect on mortality risk (0.03 vs. 0.01 vs. 0.69).

Analysis and conclusion on the effects of covariate parameters on pop-PK models are limited by the inadequate clinical parameters corrected. In addition, the external validation for the developed pop-PK model with MC simulations was not performed because this study was the first study of the pharmacokinetics of total bioactivity of AL. Since the total number of capsules administration is quite high and may influence patient’s adherence, the development of new formulation of CMC-AL is suggested. This study included the basic demographic data for population pharmacokinetic model as confounding factors (i.e., sex, age, weight, and height); however, other possible confounding factors such as smoking, genetic polymorphisms and concurrent medication were not included, which may have affected AL pharmacokinetics, particularly the metabolism process”.

## Conclusion

The developed pop-PK model with MC simulations successfully predicted clinical efficacy of CMC-AL in patients with advanced-stage iCCA. The proposed dosage regimen for phase 2B study is the once-daily dose of 2,500 mg. The application of pop-PK modelling with MC simulation, along with the appropriate cut-off values, could be applied as a promising tool to facilitate dosage prediction and selection for a subsequent clinical study, which may reduce the rate of drug development failures due to inadequate efficacy.

## Supplementary Information

Additional File 1: Table S1. OFV, AIC, BIC, and BICc values. Fig S1. Comparison predicted versus observed of plasma-concentration time profiles of total bioactivity of Atractylodes Lancea Thunb (DC) in each patient in group 1 (day 1). Fig S2. Predicted of plasma-concentration time profiles of total bioactivity of Atractylodes lancea (Thunb) DC versus observed data in group 1 (day 1). Fig S3 A virtual predictive check of plasma-concentration time profiles of total bioactivity of Atractylodes Lancea (Thunb) DC in group 1 (day 1). Fig S4. A scatter plot of residual errors of plasma-concentration time profiles of total bioactivity of Atractylodes Lancea (Thunb) DC in group 1 (day 1). Fig S5. Comparison predicted versus observed of plasma-concentration time profiles of total bioactivity of Atractylodes Lancea Thunb (DC) in each patient in group 2 (day 14). Fig S6. Predicted of plasma-concentration time profiles of total bioactivity of Atractylodes lancea (Thunb) DC versus observed data in group 2 (day 14). Fig S7 A virtual predictive check of plasma-concentration time profiles of total bioactivity of Atractylodes Lancea (Thunb) DC in group 2 (day 14).Fig S8. A scatter plot of residual errors of plasma-concentration time profiles of total bioactivity of Atractylodes Lancea (Thunb) DC in group 2 (day 14). Fig S9. Comparison predicted versus observed of plasma-concentration time profiles of total bioactivity of Atractylodes Lancea Thunb (DC) in each patient in group 2 (day 28). Fig S10. Predicted of plasma-concentration time profiles of total bioactivity of Atractylodes lancea (Thunb) DC versus observed data in group 2 (day 28). Fig S11 A virtual predictive check of plasma-concentration time profiles of total bioactivity of Atractylodes Lancea (Thunb) DC in group 2 (day 28). Fig S12. A scatter plot of residual errors of plasma-concentration time profiles of total bioactivity of Atractylodes Lancea (Thunb) DC in group 2 (day 28).

## Data Availability

The datasets used and/or analysed during the current study available from the corresponding author on reasonable request.

## Abbreviations

AIC: Akaike Information Criteria
BIC: Bayesian Information Criteria
BICc: Corrected Bayesian Information Criteria
CMC-AL: Capsule formulation standardized extract of *Atractylodes lancea* (Thunb) DC
DCR: Disease Control Rate
HR: Hazard Ratio
iCCA: Intrahepatic Cholangiocarcinoma
MC: Monte-Carlo
MIDD: Model-Informed Drug Development
NMEs: New Molecular Entities
OFV: Objective Function Value
OR: Odd Ratios
OSR: Overall Response Rate
PFS: Progression-Free Survival
Pop-PK: Population pharmacokinetics
Pop-PK/PD: Population pharmacokinetics/pharmacodynamics\
