# Supplementary material for: Population-pharmacokinetic/pharmacodynamic model of atractylodes lancea (Thunb.) DC. administration in patients with advanced-stage intrahepatic cholangiocarcinoma: a dosage prediction
Source: BMC Complement Med Ther. 2024 Nov 6;24:384. doi: 10.1186/s12906-024-04618-8 (PMC11542380; doi:10.1186/s12906-024-04618-8)
Supplement: Supplementary file 1 — Additional File 1: Table S1. OFV, AIC, BIC, and BICc values. Fig S1. Comparison predicted versus observed of plasma-concentration time profiles of total bioactivity of Atractylodes Lancea Thunb (DC) in each patient in group 1 (day 1). Fig S2. Predicted of plasma-concentration time profiles of total bioactivity of Atractylodes lancea (Thunb) DC versus observed data in group 1 (day 1). Fig S3 A virtual predictive check of plasma-concentration time profiles of total bioactivity of Atractylodes Lancea (Thunb) DC in group 1 (day 1). Fig S4. A scatter plot of residual errors of plasma-concentration time profiles of total bioactivity of Atractylodes Lancea (Thunb) DC in group 1 (day 1). Fig S5. Comparison predicted versus observed of plasma-concentration time profiles of total bioactivity of Atractylodes Lancea Thunb (DC) in each patient in group 2 (day 14). Fig S6. Predicted of plasma-concentration time profiles of total bioactivity of Atractylodes lancea (Thunb) DC versus observed data in group 2 (day 14). Fig S7 A virtual predictive check of plasma-concentration time profiles of total bioactivity of Atractylodes Lancea (Thunb) DC in group 2 (day 14).Fig S8. A scatter plot of residual errors of plasma-concentration time profiles of total bioactivity of Atractylodes Lancea (Thunb) DC in group 2 (day 14). Fig S9. Comparison predicted versus observed of plasma-concentration time profiles of total bioactivity of Atractylodes Lancea Thunb (DC) in each patient in group 2 (day 28). Fig S10. Predicted of plasma-concentration time profiles of total bioactivity of Atractylodes lancea (Thunb) DC versus observed data in group 2 (day 28). Fig S11 A virtual predictive check of plasma-concentration time profiles of total bioactivity of Atractylodes Lancea (Thunb) DC in group 2 (day 28). Fig S12. A scatter plot of residual errors of plasma-concentration time profiles of total bioactivity of Atractylodes Lancea (Thunb) DC in group 2 (day 28). [file 12906_2024_4618_MOESM1_ESM.docx]

**Supplementary material**

**Table S1.** OFV, AIC, BIC, and BICc values

|  | **Group 1 (Day 1)** | **Group 2 (Day 14)** | **Group 2 (Day 28)** |
| --- | --- | --- | --- |
| **OFV** | 566.47 | 837.42 | 741.81 |
| **AIC** | 582.47 | 853.42 | 757.81 |
| **BIC** | 586.35 | 859.09 | 762.53 |
| **BICc** | 597.34 | 870.07 | 773.31 |

**Figure S1.** Comparison predicted versus observed of plasma-concentration time profiles of total bioactivity of *Atractylodes Lancea* Thunb (DC) in each patient in group 1 (day 1).

**
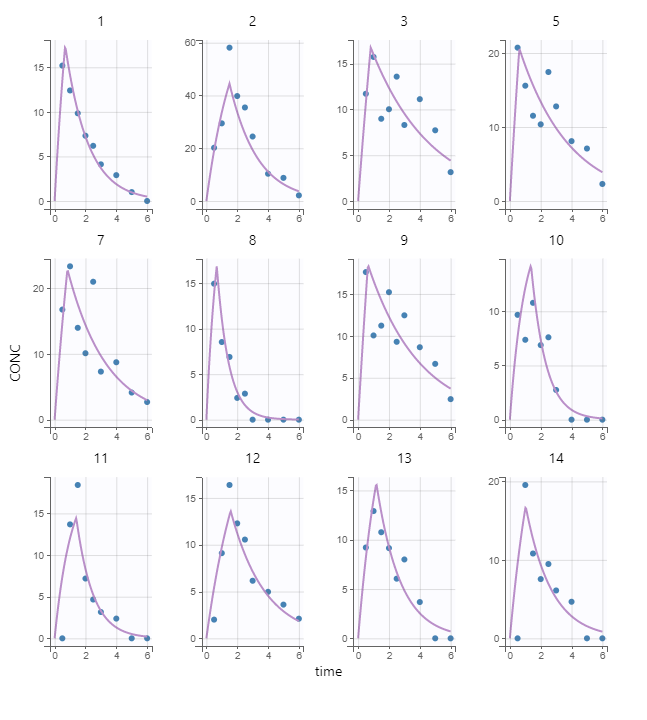
**

**Figure S2.** Predicted of plasma-concentration time profiles of total bioactivity of Atractylodes lancea (Thunb) DC versus observed data in group 1 (day 1)

**
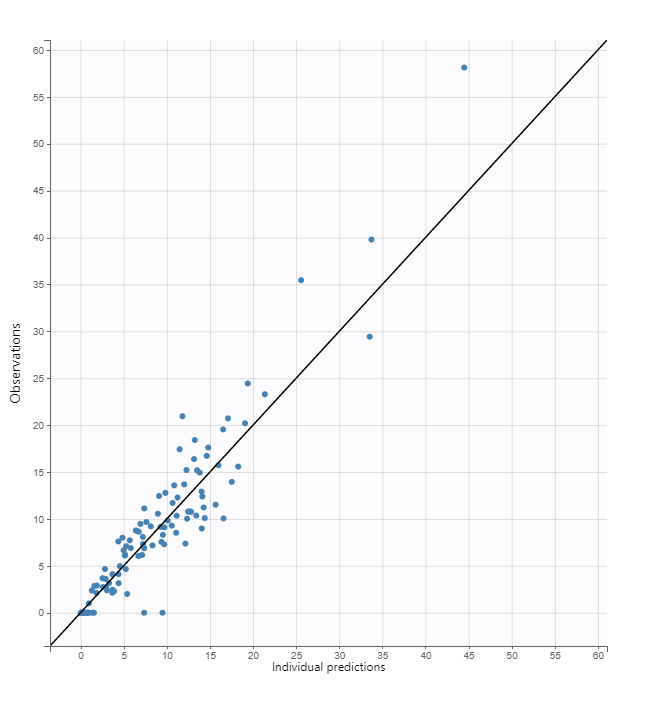
**

**Figure S3** A virtual predictive check of plasma-concentration time profiles of total bioactivity of Atractylodes Lancea (Thunb) DC in group 1 (day 1).

**
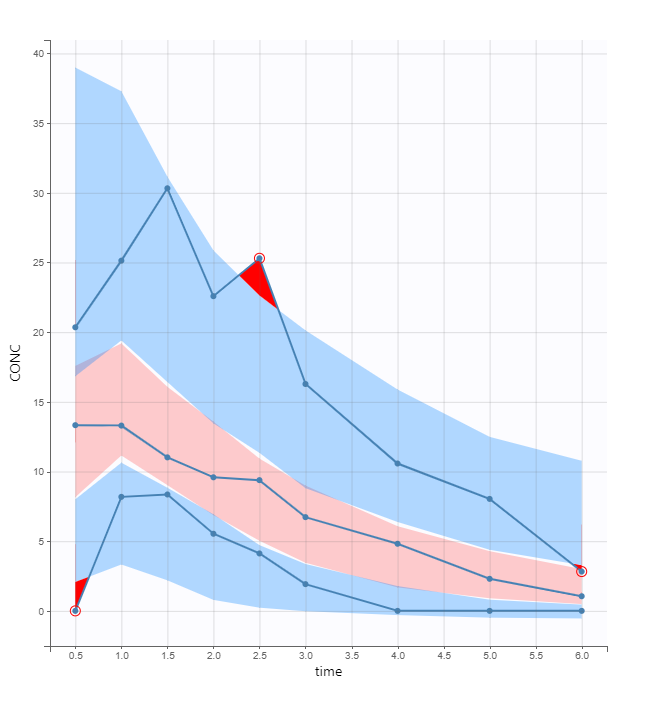
**

**Figure S4.** A scatter plot of residual errors of plasma-concentration time profiles of total bioactivity of Atractylodes Lancea (Thunb) DC in group 1 (day 1).

**
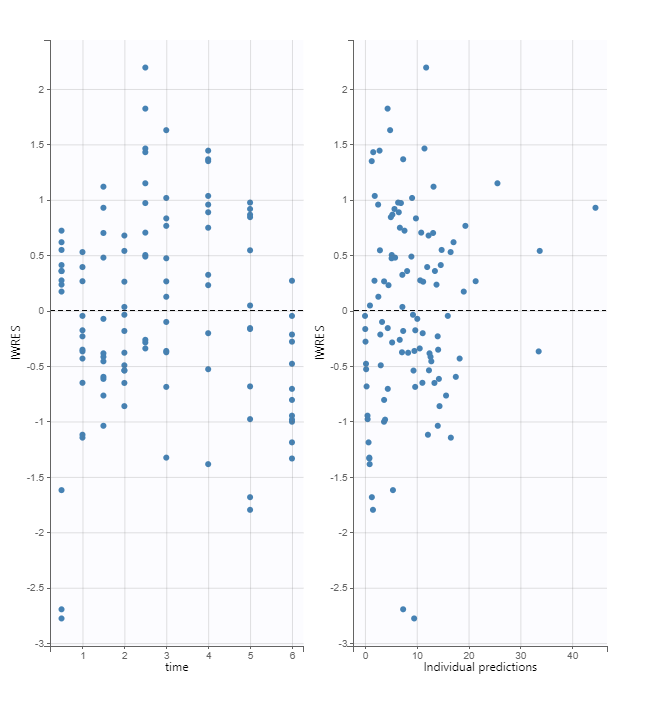
**

**Figure S5.** Comparison predicted versus observed of plasma-concentration time profiles of total bioactivity of *Atractylodes Lancea* Thunb (DC) in each patient in group 2 (day 14).

**
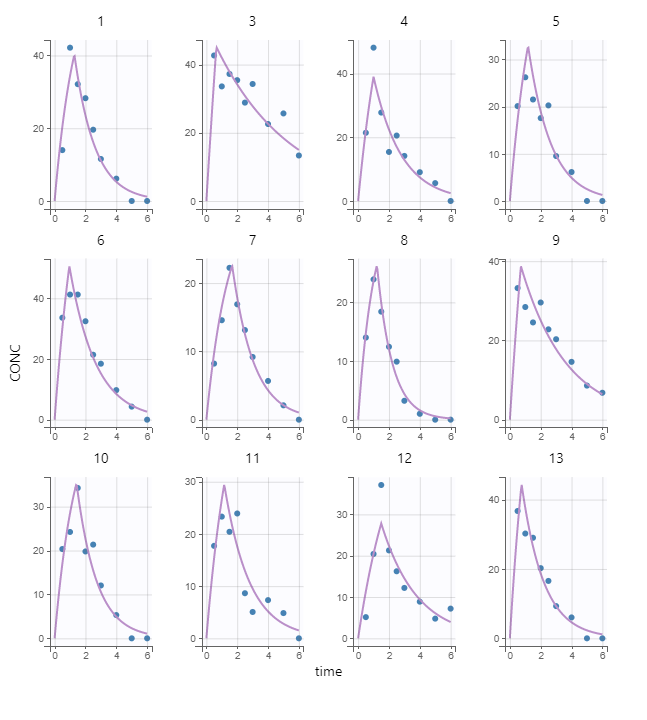
**

**
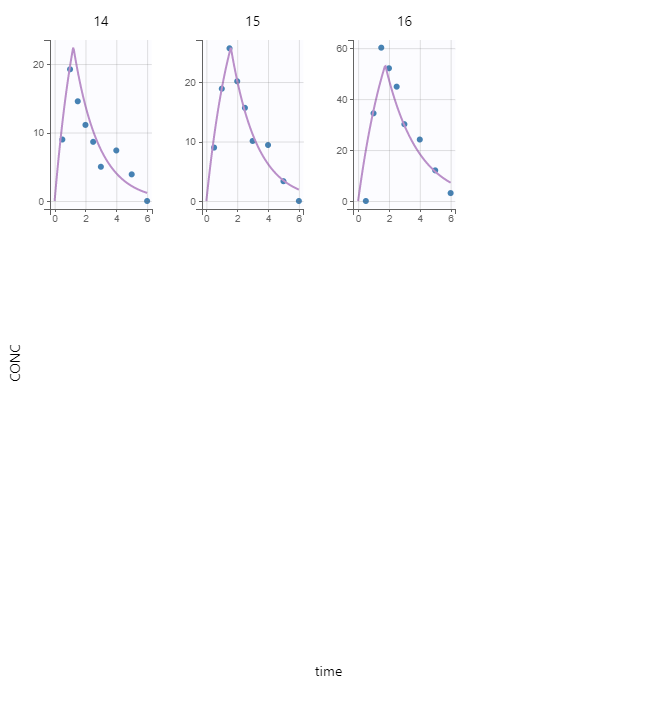
**

**Figure S6.** Predicted of plasma-concentration time profiles of total bioactivity of *Atractylodes lancea* (Thunb) DC versus observed data in group 2 (day 14)**.**

**
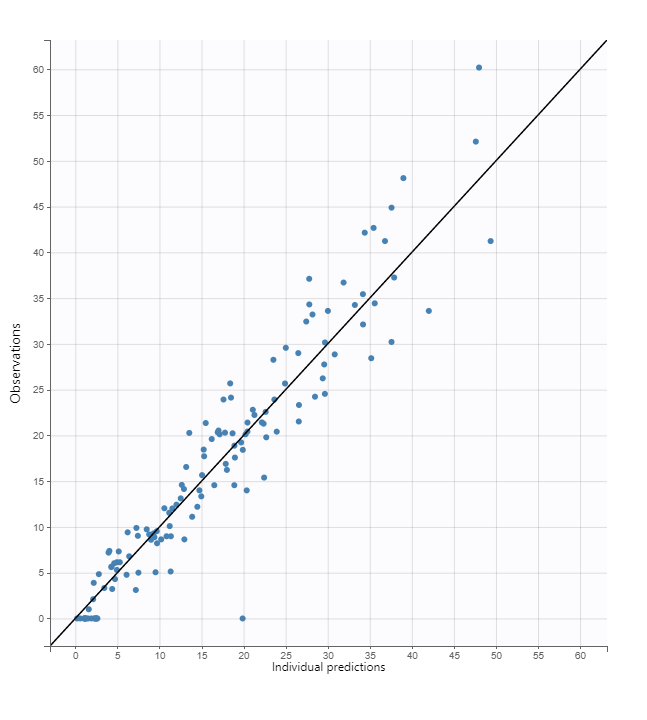
**

**Figure S7** A virtual predictive check of plasma-concentration time profiles of total bioactivity of *Atractylodes Lancea* (Thunb) DC in group 2 (day 14).

**
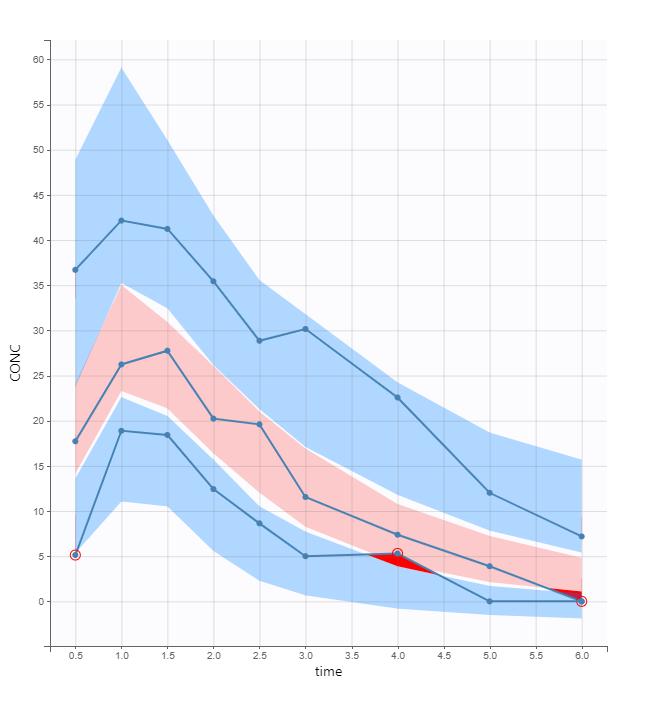
**

**Figure S8.** A scatter plot of residual errors of plasma-concentration time profiles of total bioactivity of *Atractylodes Lancea* (Thunb) DC in group 2 (day 14).

**
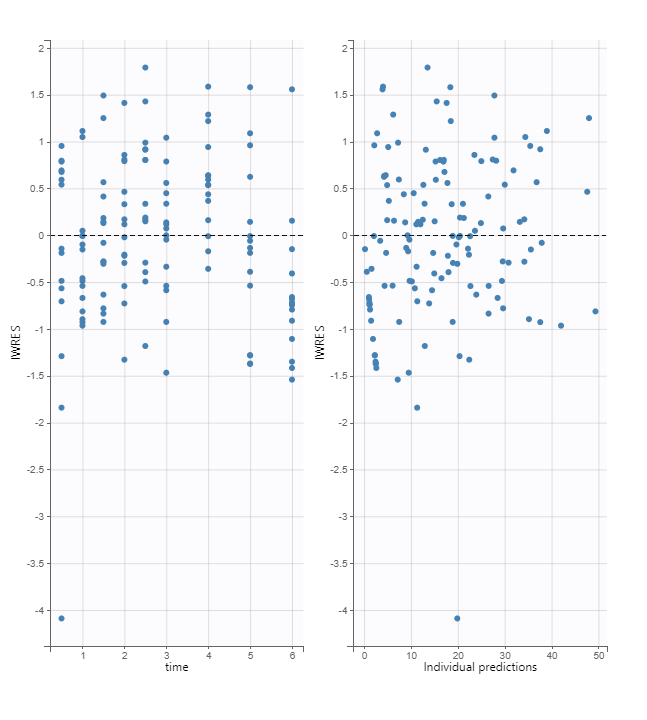
**

**Figure S9.** Comparison predicted versus observed of plasma-concentration time profiles of total bioactivity of *Atractylodes Lancea* Thunb (DC) in each patient in group 2 (day 28).

**
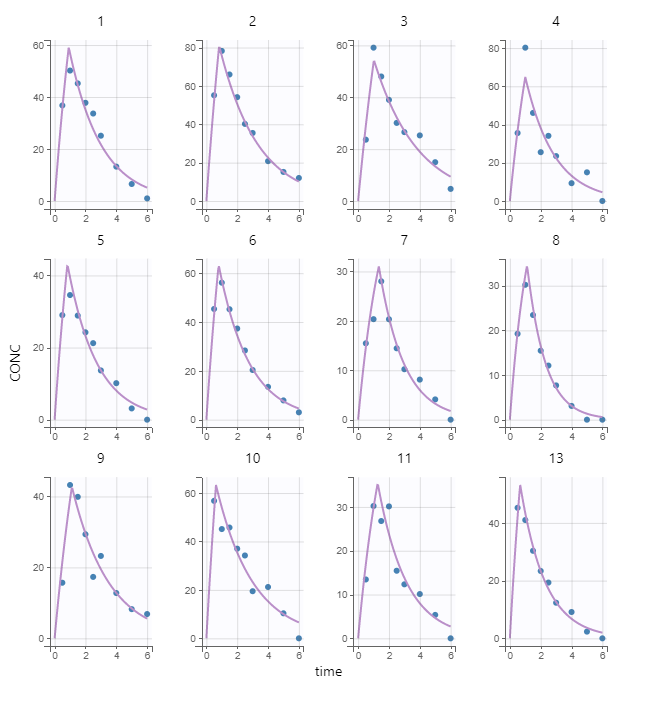

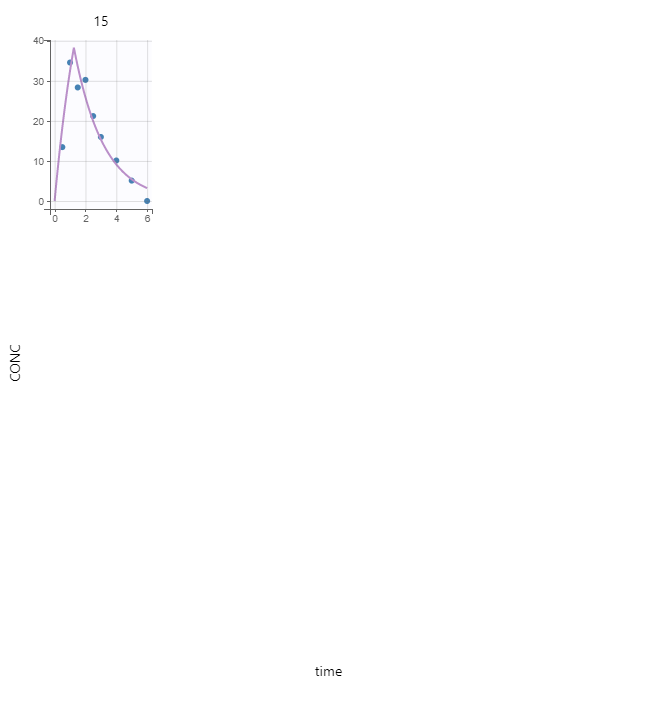
**

**Figure S10.** Predicted of plasma-concentration time profiles of total bioactivity of *Atractylodes lancea* (Thunb) DC versus observed data in group 2 (day 28)**.**

**
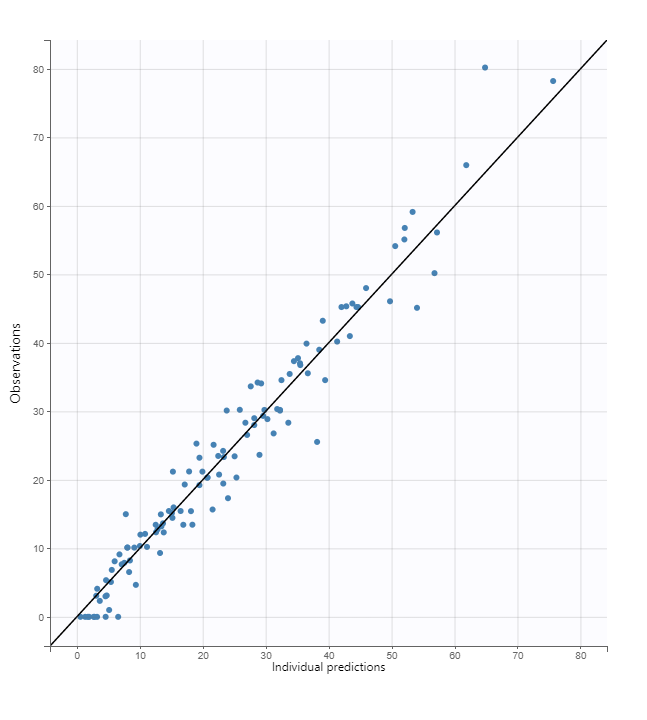
**

**Figure S11** A virtual predictive check of plasma-concentration time profiles of total bioactivity of *Atractylodes Lancea* (Thunb) DC in group 2 (day 28).

**
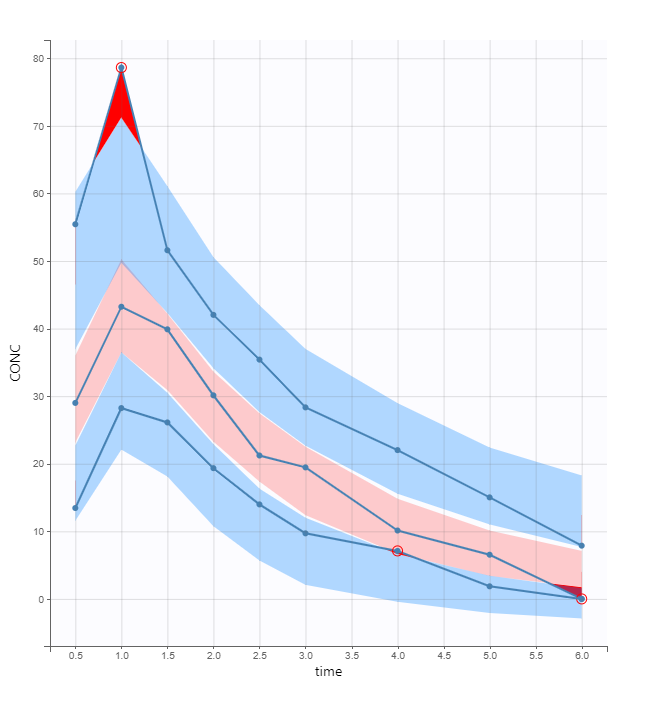
**

**Figure S12.** A scatter plot of residual errors of plasma-concentration time profiles of total bioactivity of *Atractylodes Lancea* (Thunb) DC in group 2 (day 28).

**
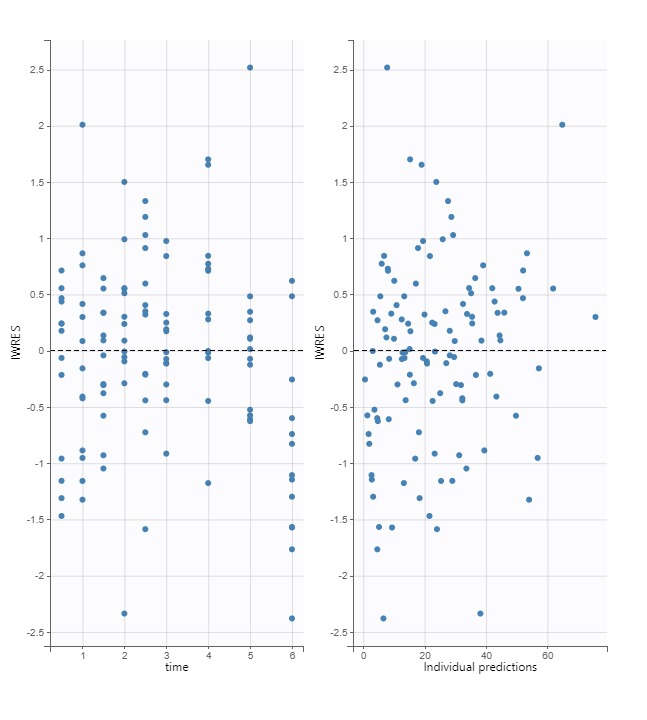
**
